# Type A Aortic Dissection of the Donor Aorta After Heart Transplantation

**DOI:** 10.1016/j.atssr.2025.09.018

**Published:** 2025-10-17

**Authors:** Sven Geurts, Aernoud T.L. Fiolet, Marish I.F.J. Oerlemans, Ronald C.A. Meijer, Linda M. de Heer

**Affiliations:** 1 Department of Cardiothoracic Surgery, University Medical Center Utrecht, the Netherlands; 2 Department of Cardiology, University Medical Center Utrecht, the Netherlands

## Abstract

Type A aortic dissection (TAAD) is a rare, but potential lethal complication post-heart transplantation (HTx) that typically affects the native aorta. This case report describes a supracoronary ascending aortic replacement in a patient who was admitted with a TAAD of the donor aorta 24 years post-HTx. This case underscores the importance of long term monitoring and management in HTx recipients, especially those with risk factors like hypertension, to prevent late aortic complications in their life after transplantation.

Long-term survival after heart transplantation (HTx) has been notably improved due to advancements in medical management and surgical techniques.^1^ However, this has also increased the incidence of late cardiovascular complications. Type A aortic dissection (TAAD) post-HTx is a rare, but potentially fatal complication and usually involves the native aorta.^2^^,^^3^ This report describes a case of TAAD of the donor aorta occurring 24 years after HTx.

A 47-year-old woman was admitted to the emergency department with acute thoracic pain radiating to the back and left arm. She underwent an orthotopic HTx 24 years earlier for end-stage heart failure due to a MYBPC3 hypertrophic cardiomyopathy. The donor heart in 2001 was from a 48-year-old female individual with a structurally normal heart who died of traumatic brain injury. The immunosuppressive regimen included cyclosporine, cellcept, and prednisolone. Our patient did not have any episodes of graft rejection in the years following. She was treated for hypertension with candesartan. Earlier therapies with angiotensin-converting enzyme inhibitors, calcium channel blockers, beta blockers, and alpha blockers were ceased due to intolerance. Notably, her office blood pressure was 130/80 mm Hg. The measurements of the aorta 21 years after the HTx still remained within normal ranges.^4^ The measurements of the aorta are presented as a mean and were as follows: aortic annulus, 21.3 mm; sinuses of Valsalva, 33.5 mm; and sinotubular junction, 30.6 mm. The measurements were assessed by 2 independent researchers blinded to each other’s measurements.

On physical examination at the emergency department the chest pain had subsided. She had a blood pressure of 150/80 mm Hg measured at both arms. The heart rate was 80 bpm, respiratory rate 24/min, and she was normoxaemic on room air. There were no systolic or diastolic heart murmurs and she was euvolemic. The electrocardiogram showed sinus rhythm with new T-wave inversion in the high lateral leads. Laboratory tests revealed elevated and rising troponin (327, 590, 600, and 800 ng/L) with normal creatine kinase levels, unchanged renal impairment (creatinine 157 μmol/L), and normal bloodcount. Chest radiography demonstrated no evidence of pulmonary congestion or infiltrates suggestive of infection, but did show a widened mediastinum.

Screening transthoracic echocardiography showed normal left and right ventricular geometry and kinetics, a moderate aortic regurgitation, and 18 mm of pericardial fluid around the left ventricle. She was admitted to the coronary care unit with an acute coronary syndrome as initial working diagnosis. A few hours after admission she developed a new episode of chest pain with ST segment elevation in the high lateral leads. An urgent coronary angiography was performed, which revealed no coronary occlusions but contrast extravasate suggestive of aortic root dissection. Subsequent computed tomography angiography of the aorta showed an aortic dissection starting at the noncoronary cusp and ending at the site of aortic anastomosis between the donor heart and native aorta (Supplemental Video). Subsequently, emergency surgery was prepared.

Cardiopulmonary bypass was performed via femoral artery and femoral vein cannulation. After careful dissection, clamping, and opening of the aorta, inspection revealed that the dissection was localized to the anastomosis between the donor and native aorta, with an intimal tear just above the sinotubular junction, predominantly on the dorsal side. It was clearly visible that the dissection stopped distally at the anastomosis, while it extended proximally behind the cusps. The root was reconstructed using bovine pericardial strips and Bioglue (Artivion) (Figure). A supracoronary ascending aortic replacement was successfully performed using a 28-mm Gelweave graft (Terumo Aortic). The patient recovered well and was discharged on day 22.

**Figure:**
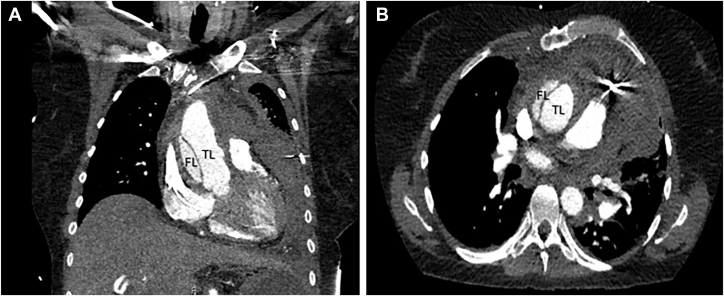
(A) A computed tomography coronal image showing the type A aortic dissection confined to the donor aorta. (B) A computed tomography transversal image showing the type A aortic dissection confined to the donor aorta. (FL, false lumen; TL, true lumen.)

Histologic examinations revealed minor atherosclerotic changes, mild medial degeneration, and no convincing indications of a (hereditary) connective tissue disorder, as well as some reactive changes with fibrosis and calcifications, most likely secondary to status post heart transplantation (HTx).

## Comment

TAAD is a rare and potentially fatal complication post-HTx. Several predisposing risk factors such as hypertension, immunosuppressive agents, and posttransplant weight gain are associated with aortic dissection.^1^^,^^5^ In a few cases the aortic dissection occurred during the early postoperative period and in most cases it occurred several years postoperatively.^2^^,^^3^ We report a TAAD 24 years post-HTx which was successfully treated with a supracoronary aortic replacement.

The diagnosis of aortic dissection in post-HTx patients is complex as its symptoms may mimic myocardial infarction, heart failure, and pericardial effusion. Moreover, the denervation of the transplanted heart may mask symptoms. The pathophysiology of the aortic dissection may also differ based on the time of onset. Early postoperative aortic dissection might be related to weakness of the aortic tissue and donor and recipient aorta mismatch. Other possibilities include postoperative infections resulting in mediastinitis with mycotic or bacterial pseudoaneurysms. Late postoperative aortic dissection is more likely caused by endothelial dysfunction due to hypertension and immunosuppressive agents. Notably, in case of dissection of the donor aorta, the native aorta can be clamped, and an open arch procedure with selective cerebral perfusion is not required.

In conclusion, we report a case of TAAD confined to the donor aorta 24 years post-HTx, successfully treated with a supracoronary ascending aortic replacement. We hypothesize that donor aortic pathology, compounded by chronic hypertension of the donor and recipient and immunosuppressive agents, may have contributed to the aortic dissection. This emphasizes the importance of long-term monitoring and blood pressure management in HTx recipients, especially those with hypertension, to mitigate late aortic complications in their life after heart transplantation.
